# A hierarchical navigation decision-making method for UAV swarms in unknown communication-constrained environments

**DOI:** 10.1371/journal.pone.0339225

**Published:** 2026-09-09

**Authors:** Huajie Xiong, Baoguo Yu, Jingkui Zhang, He Dong

**Affiliations:** 1 CETC 54th Research Institute, Shijiazhuang, China; 2 State Key Laboratory of comprehensive PNT Network and Equipment Technology, Shijiazhuang, China; Xidian University, CHINA

## Abstract

In recent years, Unmanned Aerial Vehicles (UAVs) have gradually been widely used in various fields such as regional search and disaster relief, and the development of related technologies has also experienced unprecedented growth. Compared to individual UAVs, the collaborative execution of tasks by UAV swarms has more advantages, but it is often difficult to achieve fast and accurate autonomous navigation and obstacle avoidance capabilities in unknown complex obstacle environments due to limitations in computing load and inter-UAV communication capabilities. To solve this problem, a hierarchical navigation decision-making framework for non-communication UAVs in unknown environments is proposed in this paper, which decomposes the UAV navigation planning task into an upper-layer global planning module and a lower-layer autonomous navigation and obstacle avoidance module. For the lower-layer module, an enhanced hybrid feature extraction network is designed, accompanied by a dual-stage training strategy that integrates traditional optimization methods with reinforcement learning. The upper-layer module incorporates a hybrid control strategy combining conventional search methods. Based on the ROS framework, simulation experiments for UAV swarm navigation were systematically conducted. The experimental results demonstrate that the proposed algorithm achieves autonomous navigation decision-making for multiple UAVs in complex unknown obstacle environments without relying on inter-UAV communication, showing significant advantages compared to existing approaches.

## 1 Introduction

With the development of low altitude economy and other fields, drones have become an important component of related professional applications and public service markets [1,2]. Faced with increasingly complex application environments and diverse task requirements, a single drone is limited by its mission payload, resulting in low efficiency and poor flexibility [3,4]. Therefore, the collaborative completion of aerial reconnaissance, target tracking and other tasks by clusters composed of multiple drones will become the mainstream trend in the future [5]. In addition, compared to a single drone, there are more advantages in performing autonomous navigation tasks in complex obstacle environments by drones [6,7]. On the one hand, the advantage lies in the quantity. Drones can obtain global observation information by fusing local observation information of other members in the cluster, thereby making better decisions [8]. On the other hand, due to its distributed collaborative control method, even if a drone in the cluster is damaged due to failure or third-party factors, it will not affect other drones to continue executing tasks [9,10].

The core idea of traditional navigation algorithms is to use path planning strategies to search for a safe flight trajectory from the starting point to the target point [11]. This type of method is usually applied to static or low dynamic environment, and does not require high response speed from the agent [12]. However, drones have higher requirements for obstacle avoidance and navigation in consideration of the increasingly diversified application environment, especially in densely populated and high-rise environments, which require drones to have safe, efficient, and fast response capabilities of obstacle avoidance. Therefore, obstacle avoidance and navigation technology for complex environment has become one of the main research directions in this field [13].

In recent years, multi-agent reinforcement learning (RL) of has received widespread attention as one of the solutions to intelligent control and decision-making problems in drones [14]. In the method, each agent in drones can interact with the environment and engage in cooperative and adversarial autonomous learning based on their strong ability to perceive and process complex information.

At present, there are two types of autonomous navigation technologies based on DRL: centralized and distributed [15]. The core idea of the former is to assume that all knowledge, including the intention (such as initial state and target) and workspace (such as 2D grid map) of the drone are provided to the central server to control the behavior of the cluster [16], so that collision avoidance operations can be generated by planning the optimal path. For example, Schwartz [17], Yu [18], and Tang [19] proposed a hypothesis that the strategies of other agents are fixed and unchanged. Each agent independently trains its own strategy, and all agents upload their policy gradients to the central node to update policy parameters and proceed to the next training session. This centralized approach is difficult to apply to large-scale unmanned systems, and its performance may be poor when the task complexity is high.

The core idea of the latter, the distributed approach, is a strategy at the agent level [20]. Each drone makes independent decisions while considering the observable states of other drones (such as shape, velocity, and position) as inputs, which can effectively reduce the computational processing pressure on the central server, and achieve fast and effective navigation decision-making better. For example, [21] adopted a phased training approach of traditional navigation strategy combined with Deep Deterministic Policy Gradient (DDPG), and obtained a better control strategy. This method does not focus on avoiding environmental obstacles but only on collision avoidance between drones, which can only be applied to a few fields such as drone light shows. [22] drew inspiration from the policy gradient algorithm, the intelligent drone maps its own state and the states of its neighbors to the collision free behavior space, so that it can fly from any starting point to its destination in large-scale complex environments.

However, in the face of increasingly complex task requirements and application environments, there is pressure from excessive computational load and limited cluster interaction communication of drones when executing tasks, and it may be difficult to achieve good results with traditional Deep Reinforcement Learning (DRL) based autonomous navigation methods [23,24]. To address this challenge, a hierarchical navigation decision-making method for unknown environments is proposed in this paper, which decomposes the UAV navigation planning task into an upper-layer global planning module and a lower-layer autonomous navigation and obstacle avoidance module. An improved hybrid feature extraction network with a dual-stage training strategy is designed for the lower-layer module, while a hybrid control strategy incorporating traditional search methods is developed for the upper-layer module.

Simulation results demonstrate that in various unknown complex obstacle environments, multiple UAVs trained with the proposed algorithm can successfully accomplish obstacle avoidance and navigation tasks without communication interaction, achieving higher navigation success rates and greater average reward function values compared to other existing algorithms.

## 2 Materials and methods

### 2.1 Overall framework design of algorithm

#### 2.1.1 Control model of drones.

The constraints on the environment and navigation tasks are set up as following:

- All drones are isomorphic and modeled as spheres of the same radius d, the collision threshold is $d_{r}$. If the distance between the geometric center of the drone and the geometric center of an obstacle is less than $d_{r}$, a collision is determined to have occurred.
- All drones have partially observable environmental information. At each moment t, the ith (1≤i≤N) drone can only observe partial environmental information $o_{t}^{i}$, then calculate the current collision free behavior $a_{t}^{i}$ based on the observed values, and drive oneself to move from the current position $p_{t}^{i}$ towards the target point $g^{i}$.
- There is no communication between drones. Each drone cannot directly obtain the status information or movement tendency of other drones. All drones independently calculate an action command $a_{t}$ based on the above observation information, and this command is sampled from the random strategy π shared by all drones, i.e.,$\;a_{t}\sim \pi_{\theta }(a_{t}|o_{t})$, where θ represents the parameter of the behavior strategy.
- Drones can use map construction technology to obtain surrounding information, which means that the environment can be reused after exploration.

**Fig 1:**
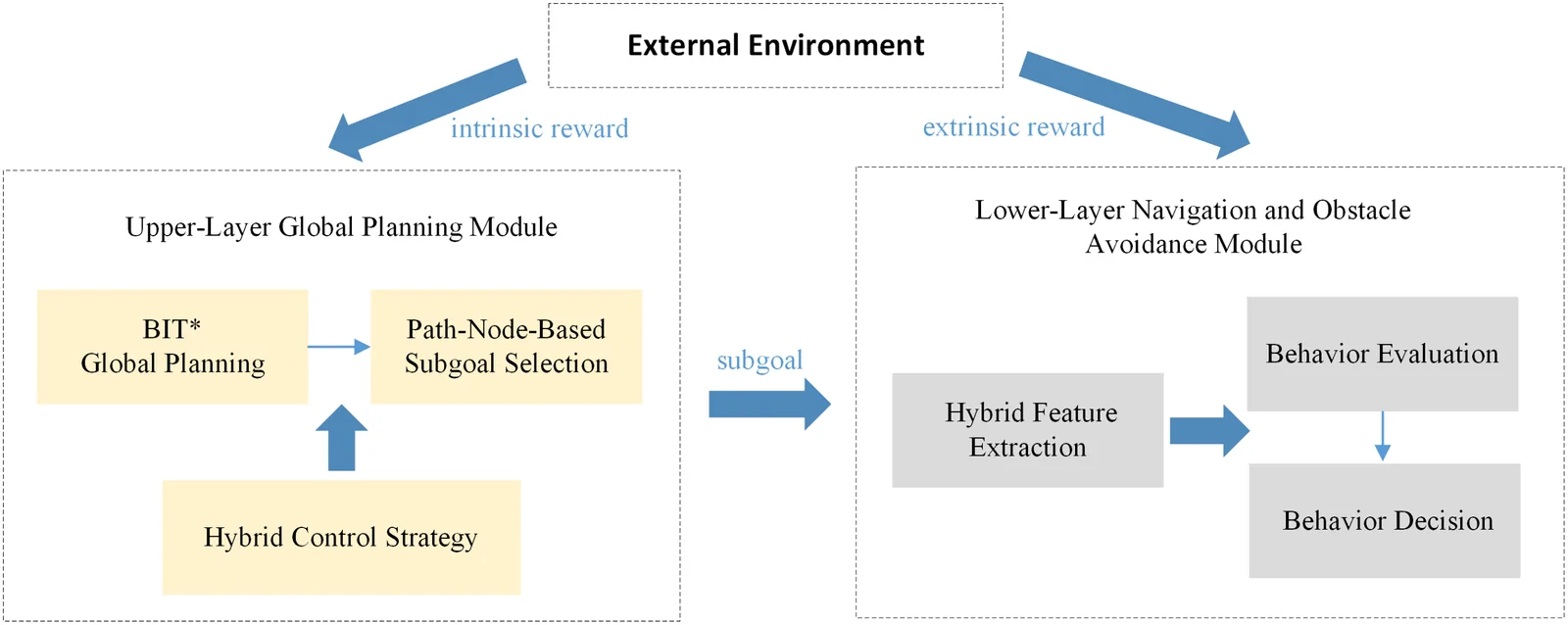
Quadrotor drones flight control system simulation.

To avoid the behavior of drones moving at high acceleration during reinforcement learning training, SO(3) [25] is used to model the drone’s motion posture. Similar to conventional flight control systems, position commands are first converted into velocity commands before being fed into the attitude controller. However, differing from standard controllers, the SO(3) controller inputs an SO(3) matrix (rather than Euler angles) to the attitude controller, and similarly receives SO(3) matrix feedback from the multi-rotor system, as shown in Fig 1.

#### 2.1.2 Hierarchical navigation decision-making task architecture design.

This paper addresses issues of continuous control and policy stability in traditional RL of UAV navigation by proposing a hierarchical reinforcement learning (HRL) navigation framework that incorporates conventional navigation control. The framework adopts a two-layer modular design that decomposing the original navigation task into upper-layer global planning and lower-layer navigation with obstacle avoidance. The upper-layer global planning module primarily functions for subgoal selection based on sampling and hybrid control strategies, outputting global path nodes; while the lower-layer obstacle avoidance navigation module employs an improved feature extraction network for local obstacle avoidance navigation, outputting local behavioral actions.

**Fig 2:**
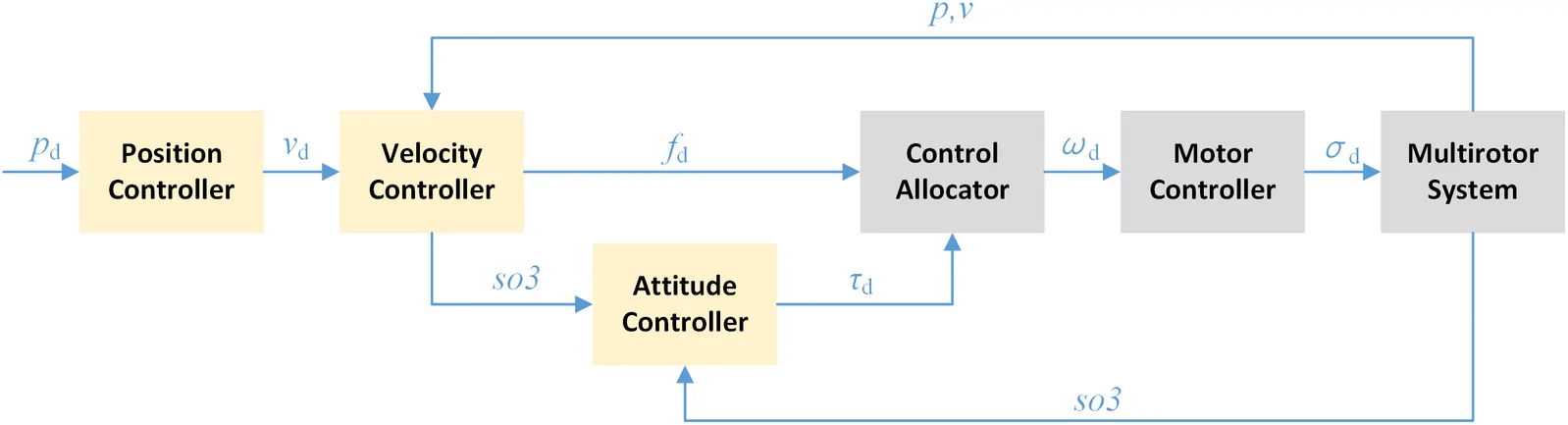
Hierarchical navigation decision-making architecture of UAVs.

Before executing each specific action, the UAV first fuses the current environmental state observation $s_{t}^{i}$ with the planned path nodes $\left\{p_{\text{1}},p_{\text{2}},...,p_{N}\right\}$, and inputs this combined information into the subgoal selector $\pi_{a}$. Through neural network mapping, it outputs the subgoal $g_{sub}^{i}$ for the current task. After acquiring the local subgoal $g_{sub}^{i}$, the UAV fuses it with the state information $s_{t}^{i}$, and processes it through the policy network of the underlying obstacle avoidance system to output the current control command $a_{t}^{i}$ for maneuvering. Once a subgoal $g_{sub}^{i}$ is determined, the underlying controller will persistently use this subgoal until either reaching the target position or until the target is reset. The UAVs repeat this process until the navigation task is completed or the current subgoal is reset. The specific framework is illustrated in Fig 2.

The reward function within this framework is also divided into two parts: intrinsic reward and extrinsic reward. The intrinsic reward corresponds to the reward function of the upper-layer global planner, while the extrinsic reward corresponds to the reward function of the lower-layer reinforcement learning controller. The specific designs of these two components are detailed in the following sections. The intrinsic reward reflects the effectiveness of the subgoal selector, whereas the extrinsic reward represents the overall navigation performance. The ultimate goal of the navigation task is to maximize the extrinsic reward.

### 2.2 Upper-layer global planning module design

The upper-layer global planning module primarily employs a RL navigation algorithm integrated with traditional path planning. This approach can provide low-frequency directional guidance for UAVs in highly dynamic environments, preventing over-reliance on the drone’s inherent RL capabilities and avoiding local optima.

#### 2.2.1 Design of a path-node-based subgoal selector.

To plan key path nodes in unknown environments and provide directional guidance for the lower-layer RL navigation algorithm, the sampling-based global planner Batch Informed Trees (BIT*) is utilized to achieve regional path search. The input of the algorithm includes the state space X, the UAV’s position$\;x_{init}$, the target position${\;x}_{goal}$, and the maximum planning time T, while the output is a series of minimum-cost path nodes within the obstacle-free space [26]. The entire problem is addressed by maintaining a randomly expanding tree (V,E), where V⊆X, and E represents the edges between points in V.

The state space of the upper-layer subgoal selector consists of the current observation value $o_{t}^{l}\;$from the depth sensor of UAVs, the current velocity value $o_{t}^{v}$ of the drone, and the five closest nodes $\left\{p_{\text{1}},p_{\text{2}},p_{\text{3}},p_{\text{4}},p_{\text{5}}\right\}$ from the current path planning, ensuring that these nodes are within the UAV’s perceptual range. If there are fewer than 5 nodes, they are interpolated at equal intervals; if there are more than 5 nodes, only the 5 closest nodes are selected. Additionally, to maintain consistency in the controller’s coordinate system and eliminate the neural network’s sensitivity to absolute distances, this paper converts all these nodes into relative positions in the UAV’s polar coordinate system [27].

The reward function of the subgoal selector consists of three components:


$$\begin{array}{c} r_{sub}=r_{collision}+r_{bit}+r_{safe} \end{array}$$ (1)

In the above equation, $r_{collision}$ represents the collision penalty, with $r_{collision}=-\text{1}\text{0}$; $r_{bit}$ represents the deviation penalty from the global path, with $r_{bit*}=-\text{0}.\text{5}d_{bit*}$, here $d_{bit*}$ represents the distance between the UAV’s current position and the next node planned by BIT*; $r_{safe}$ represents the safe distance penalty, if the distance to the nearest obstacle falls below the safety threshold, i.e., $d_{obs}\leq d_{r}$, $r_{safe}=-\text{2}\left(\text{0}.\text{5}-d_{obs}\right)$, in all other cases, $r_{safe}=\text{0}$.

The subgoal selector employs an alternating update strategy for the policy network $\pi_{\theta }$ and the value network $Q_{\omega }$ to maximize cumulative rewards. Given the current state-action value function Q(s,a;ω), the objective function is defined as:


$$\begin{array}{c} J(\theta )=E\left[Q\left(s,\pi (s;\theta );\omega \right)\right] \end{array}$$ (2)

The state observation value s is used to compute the gradient of the objective function with respect to the actions, and gradient ascent is employed to maximize J(θ). The parameters θ of the policy network can be updated as follows, where βis the learning rate:


$$\begin{array}{c} \theta \leftarrow \theta +\beta \nabla_{\theta }\pi (s;\theta )\cdot \nabla_{a}\;Q\left(s,\hat{a};\omega \right){|}_{\hat{a}=\pi (s;\theta )} \end{array}$$ (3)

The value network is updated by using the actual observed reward r from each training round to make the predicted value $\hat{Q}(s,a)$ progressively approach the true value function Q(s,a;ω). Therefore, the parameters of the value network are updated as follows, where α is the learning rate:


$$\begin{array}{c} \begin{array}{c} {\hat{y}}_{t}=r_{t}+\gamma \cdot \hat{q}\left(s_{t+\text{1}},\pi \left(s_{t+\text{1}};\theta \right);\omega \right)\\ L=\frac{\text{1}}{\text{2}}\left[\hat{q}\left(s_{t},a_{t};\omega \right)-{\hat{y}}_{t}\right]\\ \omega \leftarrow \omega -\alpha \nabla_{\omega }L(\omega ) \end{array} \end{array}$$ (4)

#### 2.2.2 Hybrid control strategy for UAVs.

The hybrid control decision-making approach that combines reinforcement learning with traditional control methods can accelerate network convergence and enhance the drone’s movement efficiency in certain specific scenarios.

The following configuration are adopted in this paper:

- Conservative strategy${\;policy}_{safe}$
- When the distance between the UAVs and the nearest obstacle is less than a certain threshold $d_{danger}$, the scenario is considered dangerous, as collisions are highly likely to occur. In such case, a conservative strategy of reducing perception data and decelerating must be executed.
- Aggressive strategy${\;policy}_{PID}$
- When the distance between the UAVs and the nearest obstacle is greater than a certain threshold$\;d_{open}$, the scenario is considered open. In such cases, the PID control strategy should be activated.
- Normal strategy${\;policy}_{RL}$
- When the distance between the UAVs and the nearest obstacle falls between $d_{danger}$ and$\;d_{open}$, the scenario is considered normal, and the predefined strategy should be followed [28].

A state estimator for the UAV is also designed in this paper. This module primarily utilizes the UAV’s velocity $v_{t}$ and position $p_{t}$ at time t, and predicts the UAV’s position $p_{t+\Delta t}$ and velocity $v_{t+\Delta t}$ at time t+Δt based on a future time interval Δt, as illustrated in the figure below. The output of this module is incorporated as additional auxiliary perception into the reinforcement learning network of the lower-layer navigation and obstacle avoidance module discussed below.

### 2.3 Lower-layer navigation and obstacle avoidance module design

The lower-layer navigation and obstacle avoidance module primarily employs the RL algorithm with an improved feature extraction network and a dual-stage training strategy, enabling the UAV to achieve autonomous navigation and obstacle avoidance capabilities under non-interactive conditions.

#### 2.3.1 Reward function design.

The optimization objective of RL is to maximize the cumulative reward value. As an important component of RL, immediate reward R is an important reference indicator for distinguishing the behavior and states of drones. The method based on Artificial Potential Field (APF) was proposed to improve the reward function in continuous space. The main calculation idea is to use gradient descent to find the path with the lowest potential energy in the entire potential field space. The common approach is to construct an attractive field using the target location, and construct a repulsive field based on the position of obstacles. The formula for calculating the attractive field is as follows [29].


$$\begin{array}{c} U_{att}(p)=\left\{ \begin{array}{c} \frac{\text{1}}{\text{2}}\xi d^{\text{2}}\left(p-p_{g}\right),\;d\left(p,p_{g}\right)\leq r_{g}\\ r_{g}\xi d\left(p-p_{g}\right)-\frac{\text{1}}{\text{2}}\xi {\left(r_{g}\right)}^{\text{2}},\;d\left(p,p_{g}\right)>r_{g} \end{array}\right. \end{array}$$ (5)

In the above equation, ξ is the attractive gain parameter, $d\left(p,p_{g}\right)$ is the distance between the position of the drone and the goal, $r_{g}$ is the range of influence of the attractive force. The formula for calculating the repulsive field is as follows.


$$\begin{array}{c} U_{rep}(p)=\left\{ \begin{array}{c} \frac{\text{1}}{\text{2}}\eta {\left(\frac{\text{1}}{d\left(p,p_{o}\right)}-\frac{\text{1}}{r_{o}}\right)}^{\text{2}},\;d\left(p,p_{o}\right)\leq r_{o}\\ \text{0},\;d\left(p,p_{o}\right)>r_{o} \end{array}.\right. \end{array}$$ (6)

In the above equation, η is the repulsive gain parameter, $d\left(p,p_{o}\right)$ is the distance between the position of the drone and the recent obstacle, $r_{o}$ is the range of influence of the repulsive force. The overall potential field is the sum of the attractive and repulsive field. Add the above artificial potential field effect to the reward for each step of the movement of drones.

- Attraction reward function
- The attraction reward function is designed to guide the drones to move towards the goal position, as shown in the following equation.

$$\begin{array}{c} F_{t}^{att}=\xi d(p_{t-\text{1}},p_{g}) \end{array}$$ (7)

$$\begin{array}{c} r_{t}^{att}=F_{t-\text{1}}^{att}-F_{t}^{att} \end{array}$$ (8)

- The equation means that no matter where the drone is located, as long as it moves towards the goal direction, it can obtain stable benefits, thereby the drone is driven to reach the final goal position in order to obtain maximum benefits.
- Repulsion reward function
- The repulsion reward function is to prevent the drone from colliding with obstacles, which is as follows.

$$\begin{array}{c} F_{t}^{rep}=\eta (\frac{\text{1}}{r_{o}}-\frac{\text{1}}{d\left(p_{t-\text{1}},p_{o}\right)})\frac{\text{1}}{d^{\text{2}}\left(p_{t-\text{1}},p_{o}\right)}\nabla d\left(p_{t-\text{1}},p_{o}\right) \end{array}$$ (9)

$$\begin{array}{c} r_{t}^{rep}=\left\{ \begin{array}{c} @c\left\|F_{t-\text{1}}^{rep}\right\|-\left\|F_{t}^{rep}\right\|,\;d_{r}\leq d\left(p_{t},p_{o}\right)\leq r_{o}\\ \text{0},\;otherwise \end{array}\right. \end{array}$$ (10)

- In the above equation,$\;d_{r}$ is the safety distance of drones, which also serves as the lower limit of the repulsive influence threshold, when the distance between the drone and the nearest obstacle is less than the threshold, it can be determined that the drone has collided.
- Smoothness reward function
- Due to the continuous spatial sampling characteristics of reinforcement learning, it is difficult to ensure the smoothness of the motion of drones, which requires punishment for changes of drones in motion direction.

$$\begin{array}{c} r_{t}^{ori}=\left\{ \begin{array}{c} @c\delta \left|\theta_{t}-\theta_{t-\text{1}}\right|,\;\left|\theta_{t}-\theta_{t-\text{1}}\right|\geq \theta_{min}\\ \text{0},\;otherwise \end{array}\right. \end{array}$$ (11)

- In the above equation, δ is the directional penalty coefficient. $\theta_{t}\;$and $\theta_{t-\text{1}}$ are the velocity direction of the drone at the current and previous moments.
- Inertness reward function
- At the beginning of the experiment, the drones are still in the exploratory stage and is likely to circle in place, so a relatively small negative penalty needs to be given at each step of drones.

$$\begin{array}{c} r_{t}^{step}=R_{step} \end{array}$$ (12)

In addition, the goal reach rewards and collision penalties in sparse reward are retained as following.


$$\begin{array}{c} r_{t}^{rea}=\left\{ \begin{array}{c} @cR_{reach}\;,d\left(p,p_{g}\right)<d_{r}\\ \text{0}\;,\;otherwise \end{array}\right. \end{array}$$ (13)

$$\begin{array}{c} r_{t}^{col}=\left\{ \begin{array}{c} @cR_{collision\;},\;d\left(p,p_{o}\right)<d_{r}\\ \text{0}\;,\;otherwise \end{array}\right. \end{array}$$ (14)

In the above equation,$\;d_{r}$ is the safe radius of drones. $R_{reach}$, $R_{collision}$ and $R_{step}$ are all pre-set constants.

In summary, in order to solve the sparse reward problem in navigation tasks of drones, the reward function designed in the article is divided into reach reward $r_{t}^{rea}$, collision penalty $r_{t}^{col}$, attraction reward $r_{t}^{att}$, repulsion penalty $r_{t}^{rep}$, smoothness reward $r_{t}^{ori}$, and inertness penalty $r_{t}^{step}$, the overall reward function is as follows.


$$\begin{array}{c} r_{t}=r_{t}^{rea}+r_{t}^{col}+r_{t}^{att}+r_{t}^{rep}+r_{t}^{ori}+r_{t}^{step} \end{array}$$ (15)

#### 2.3.2 UAV navigation behavior strategy based on improved hybrid feature extraction.

Due to the focus of the article on solving navigation problems of drones under non-communication conditions, it can lead to a large amount of redundant information when the environment becomes complex, and traditional neural networks are usually unable to adapt to this situation well. To solve this problem, a new feature extraction network was designed in the article, which is assigned different weights depend on different information features, in order to obtain more accurate observation inputs and improve task success rates [30].

The feature extraction network is improved based on attention mechanism. A feature fusion mechanism combined with soft attention and hard attention is adopted according to the large observation value of obstacle avoidance navigation of drones in unknown environments. The core idea is first to convert the LIDAR observation data obtained at each moment to one-dimensional vector $q\in R^{\text{3}m}$, then arrange it into a sequence and pass it through an embedding layer to obtain the processed data $\left\{{\hat{l}}_{\text{1}},{\hat{l}}_{\text{2}},\ldots ,{\hat{l}}_{\frac{\text{512}}{m}}\right\}$, and the calculation method of the features of the data is to map it to a high-dimensional feature space through an adaptive feature extraction network.

**Fig 3:**
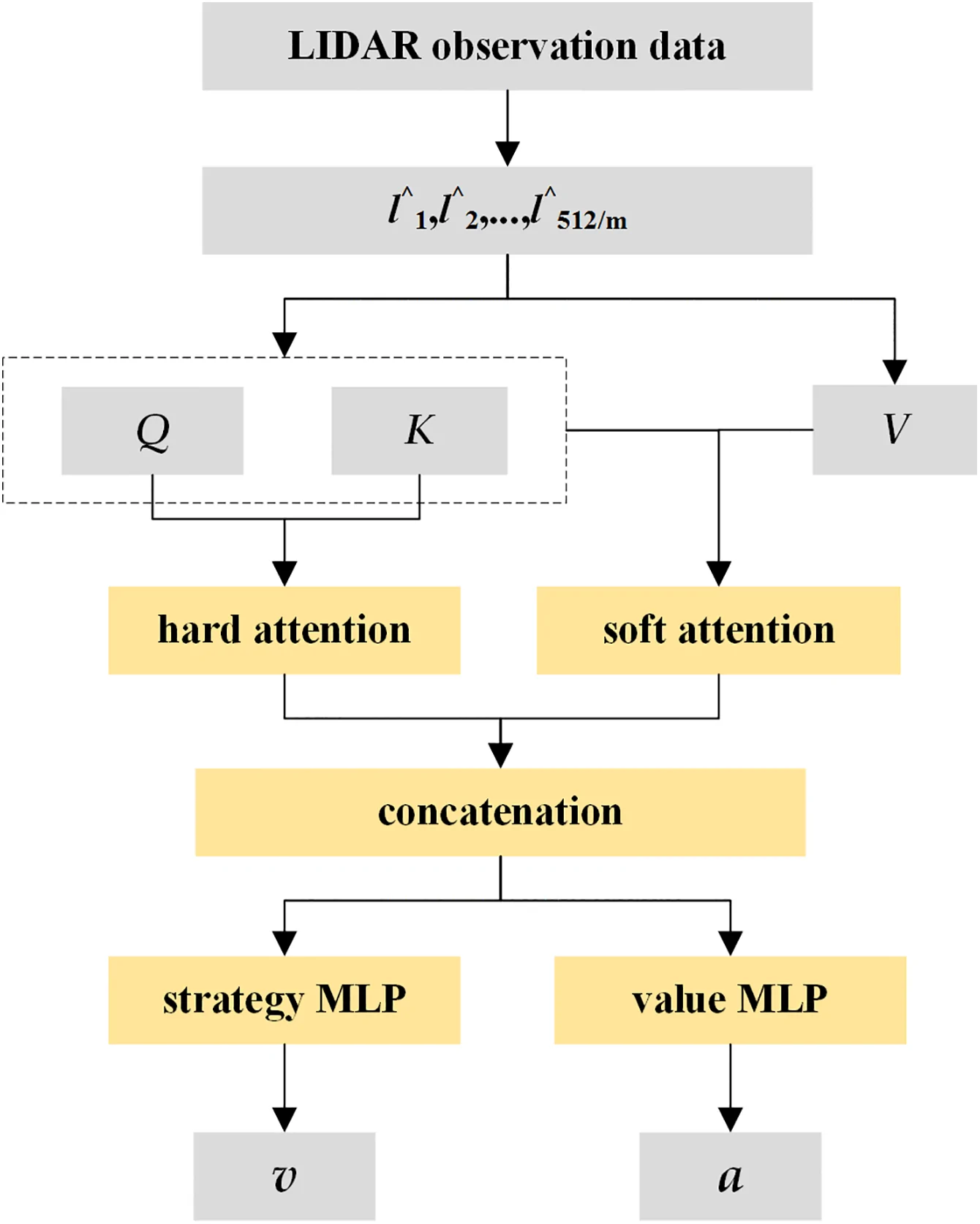
The specific steps of the improved hybrid feature extraction network.

The specific steps of the feature extraction network are as follows in Fig 3.

The query matrix $Q=\left[q_{1},q_{2},\ldots ,q_{n}\right]$, the key matrix $K=\left[k_{1},k_{2},\ldots ,k_{n}\right]$ and the value matrix $V=\left[v_{1},v_{2},\ldots ,v_{n}\right]$ are based on the observation input $X=\left[{\hat{l}}_{\text{1}},{\hat{l}}_{\text{2}},\ldots ,{\hat{l}}_{\frac{\text{512}}{m}}\right]$. The calculation formula for Q, K and V are as follows.


$$\begin{array}{c} \begin{array}{c} Q=W_{q}\cdot X\\ K=W_{k}\cdot X\\ V=W_{v}\cdot X \end{array} \end{array}$$ (16)

In the above equation, $W_{q}$, $W_{k}$ and $W_{v}$ are the parameter matrices of the linear transformation. The calculation of soft attention features is as follows.


$$\begin{array}{c} f\left(q_{i},k_{j}\right)=q_{i}\cdot {k_{j}}^{T}/\sqrt{d} \end{array}$$ (17)

$$\begin{array}{c} \begin{array}{c} s_{i}=\sum_{j=\text{1}}^{n}softmax\left(f\left(q_{i},k_{j}\right)\right)v_{j}\\ S=\left[s_{1},s_{2},\ldots ,s_{n}\right] \end{array} \end{array}$$ (18)

In the above equation, d is the length of $q_{i}$ and $k_{j}$. The calculation of hard attention features is as follows.


$$\begin{array}{c} \begin{array}{c} h_{i}={argmax}_{j}\left(softmax\left(f\left(q_{i},k_{j}\right)\right)\right)\\ H=\left[h_{1},h_{2},\ldots ,h_{n}\right] \end{array} \end{array}$$ (19)

In the above equation, ${\text{argmax}}_{j}$ represents the location of the maximum value in dimension of j.

The adaptive fusion features of each drone are obtained by convolution of soft attention features S and hard attention features H. These features contain various states such as depth information of multiple locations and velocity change information of the environment, enabling drones to avoid collisions in these locations. The final behavior instruction a is obtained by the output and parameters of the policy network through the Gaussian random sampling function, and the final state value v is exported directly by the value network.

#### 2.3.3 The dual-stage training strategy for RL based on ORCA-PSO.

Generally, machine learning-based approaches for multi-drone navigation tasks require a prior knowledge to accelerate the learning process, such as existing collision avoidance control algorithms to pre-establish knowledge domains. The Optimal Reciprocal Collision Avoidance (ORCA) algorithm is currently one of the most widely used distributed collision avoidance algorithms for multi-agent systems. By utilizing the velocity information of moving agents to construct obstacle profiles, the algorithm achieves inter-agent collision avoidance within a defined time frame [31].

But the ORCA algorithm requires each agent to obtain information such as the shape, current position, and optimal velocity of other agents in every computation cycle. This characteristic makes it unsuitable for the non-communication navigation scenarios of UAVs addressed in this study. To solve the problem, the study pro-poses a primary control strategy based on the fusion algorithm of ORCA and improved particle swarm optimization (PSO), which serves as the initial solution input for the enhanced neural network. The strategy’s generalization capability is further strengthened through reinforcement learning [32].

The traditional PSO is to find the optimal particle position based on iterative formulas under given inter-agent distance constraint, range constraint and so on, which serves as the control output for the agent. In contrast, distributed multi-agent control strategies such as ORCA take velocity vectors as input for computation and output the optimal collision-avoidance velocity for the agent. Therefore, the velocity obstacle boundary from ORCA can be incorporated as a constraint term in the evaluation function of PSO. By refining the iterative strategies for particle velocity and position updates, the algorithm can be adapted to meet the requirements of distributed decision-making in multi-agent systems.

Transforming the velocity and position information of the drones from the position coordinate system to the velocity coordinate system, the collision-avoidance set for drone i and j under ORCA is denoted as


$$\begin{array}{c} {{ORCA}^{\tau }}_{i|j}=\left\{\vec{v}|\left(\vec{v}-\left({\vec{v}}_{opt}+\frac{\text{1}}{\text{2}}\vec{u}\right)\right),\vec{n}\geq \text{0}\right\} \end{array}$$ (20)

The equation represents the velocity within the half-plane that satisfies the boundary condition passing through ${\vec{v}}_{opt}+\frac{\text{1}}{\text{2}}\vec{u}$ and oriented toward $\vec{u}$, which is the shortest velocity vector starting from $\vec{v}i_{opt}-\vec{v}j_{opt}$ and pointing outside the collision zone ${{VO}^{\tau }}_{i|j}$. The optimal critical obstacle avoidance velocity for drone iand j satisfies the following condition


$$\begin{array}{c} \vec{v}i_{opt}-\vec{v}j_{opt}+\underset{v\in {ORCA}^{\tau }i|j}{argmin}\left\|\vec{v}-\left({\vec{v}}_{i}-{\vec{v}}_{j}\right)\right\|\notin {{VO}^{\tau }}_{i|j} \end{array}$$ (21)

The iteration of the PSO requires an evaluation function to determine the search direction. Generally, the evaluation function of the PSO typically includes the mission constraint function and dynamic constraint function of drones regarding the navigation problem of drones. Incorporate the optimal obstacle avoidance velocity constraint as a constraint term in the evaluation function of the PSO, considering practical application scenarios, the evaluation function can be expressed as


$$\begin{array}{c} minF=\rho_{\text{1}}\left(f_{\text{1}}-D_{max}\right)+\rho_{\text{2}}\frac{\text{1}}{f_{\text{2}}-R_{min}}+\rho_{\text{3}}\frac{\text{1}}{f_{\text{3}}-S_{min}} \end{array}$$ (22)

In the above equation, $f_{\text{1}}$ represents the total flight distance of each drone, $f_{\text{2}}$ represents the total turning radius of each drone, $f_{\text{3}}$ represents the total spacing between all drones based on the optimal critical obstacle avoidance velocity. $D_{\text{max}}$is the maximum flight range constraint, $R_{\text{min}}$ is the minimum turning radius constraint,$\;S_{\text{min}}$ is the minimum inter-drone spacing constraint. $\rho_{\text{1}},\;\rho_{\text{2}}\text{ and }\rho_{\text{3}}$ are the weighting factors, their sum equals 1, with specific values determined by actual conditions.

**Fig 4:**
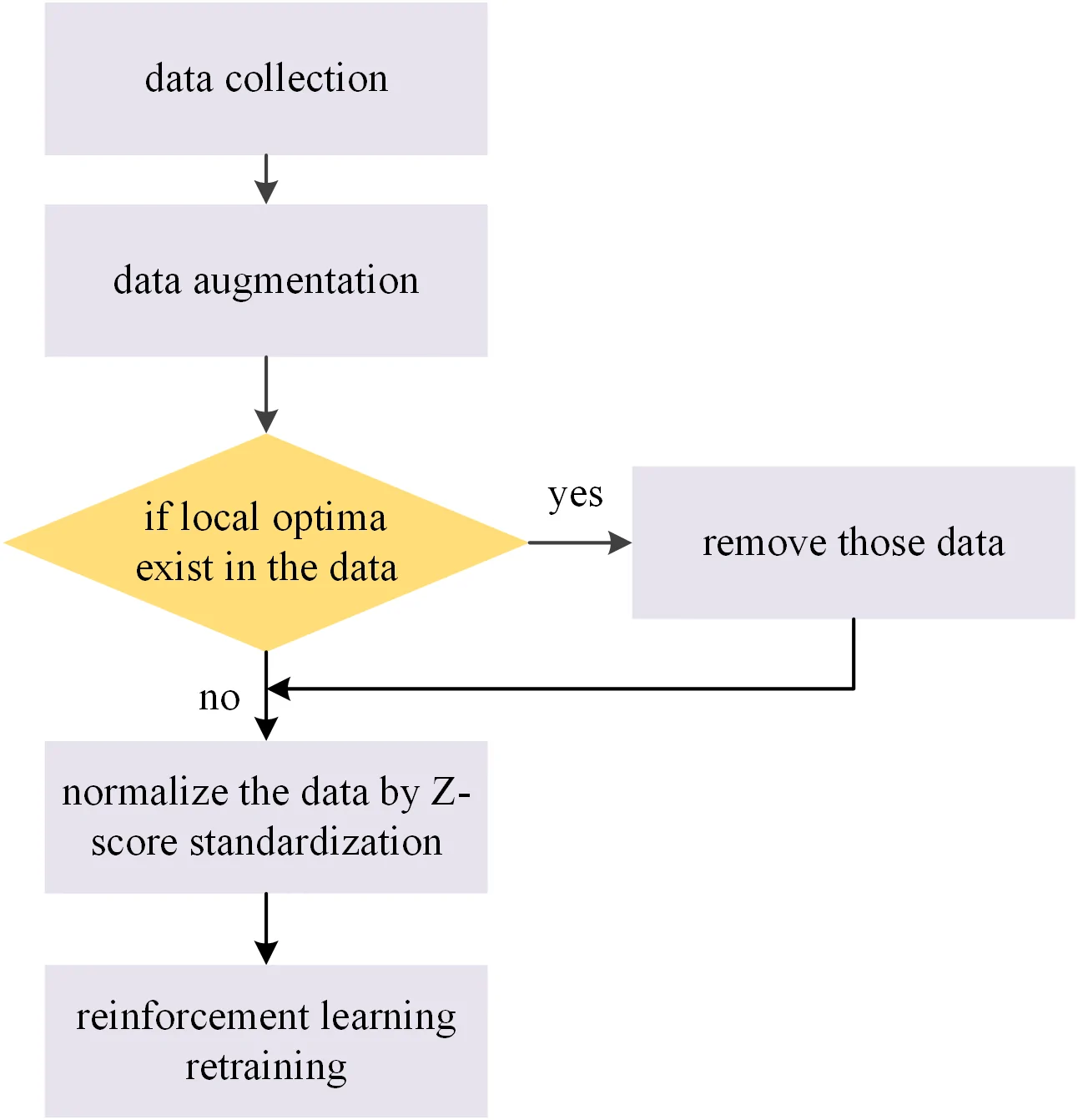
The enhanced dual-phase training strategy.

The dual-stage training strategy designed in this study serves as: the improved ORCA-PSO collision avoidance strategy introduced earlier is used to provide a high-quality initial solution for reinforcement learning, and reinforcement learning is employed to enhance its generalization capability. The main process is as follows in Fig 4.

The improved ORCA-PSO strategy is deployed in a multi-UAV system to collect data during the movement of each drone. The data requires data augmentation (e.g., adding noise) to enhance the model’s generalization capability. After filtering out these data points that adversely affect training results, the Z-score normalization on the data is performed to obtain uniformly distributed and dimensionless data. Finally, the improved ORCA-PSO control commands are directly used as labels to compute the loss function with the output of the reinforcement learning policy. This dual-phase learning method can improve the robustness and convergence speed of the algorithm.

## 3 Results

To verify the effectiveness and superiority of the algorithms proposed in this article, the algorithm was applied to several typical navigation conditions in this section, and algorithm comparisons were conducted to test the algorithm effects in different conditions and tasks.

### 3.1 Navigation algorithm experimental configuration and indicators setting

#### 3.1.1 Hardware/software configuration and evaluation indicators of algorithms.

To verify the applicability and effectiveness of the algorithm proposed in this paper, this section conducts comparative algorithm tests on several typical structured navigation scenarios, and validates the algorithm’s performance in unstructured scenarios on a simulated onboard embedded system.

- Hardware Configuration:
  1) Desktop-class system
  CPU: Intel Core i7-13620H
  2) GPU: NVIDIA GeForce GTX 1660 Super, Memory: 32GB RAM
  Simulating Onboard Embedded System
  Jetson AGX Orin 64GB
- Software Configuration:
- Ubuntu 20.04; python 3.8; pytorch 1.12; ROS Noetic
- The existing research results were referenced in the article and the following indicators to evaluate the performance of the algorithm were used. In this paper, unless otherwise specified, all results are calculated by averaging over 100 test runs.
- Success rate
- Success rate refers to the probability of reaching the goal position without collision. This indicator is used to reflect the obstacle avoidance ability of the strategy.
- Timeout rate
- The timeout rate refers to the proportion of episodes terminated due to failure to complete the task within the maximum allowed steps. This indicator is generally used to evaluate the decision-making efficiency of agents and the feasibility of the task.
- Average reward
- Average reward refers to the average reward value of a single task after model training is completed. This indicator is used to determine the superiority or inferiority of a strategy in the entire task.

#### 3.1.2 Navigation environment of drones.

In order to test the navigation performance of drones under limited information, the side length of drones d is 0.5 meters, and the safety radius $d_{r}$ is 0.4 meters, the maximum sensing range of the drone’s LiDAR is limited to 6 meters, the minimum range is 0.3 meters, and the sensing frequency is 10 Hz. The update frequency of the odometry of drones is 100 Hz. The inertia reward constant $R_{step}=-\text{0}.\text{1}$, the goal reach reward constant $R_{reach}=\text{1}\text{0}$, and the collision penalty constant $R_{collision}=-\text{1}\text{0}$; The attractive gain parameter ξ=0.6 , the repulsive gain parameter η=1.2 , the lower limit of the repulsive influence threshold $r_{o}^{*}$ is 1.5 meters, the directional penalty coefficient δ=0.05 ; The maximum flight range constraint $D_{\text{max}}$ is 50 meters, the minimum turning radius constraint $R_{\text{min}}$ is 0.5 meters, the minimum inter-drone spacing constraint$\;S_{\text{min}}$ is 0.35 meters, the weighting factors $\rho_{\text{1}}=\text{0}.\text{6},\;\rho_{\text{2}}=\text{0}.\text{1},\;\rho_{\text{3}}=\text{0}.\text{3}$. The number of drones is configured according to specific environment.

**Fig 5:**
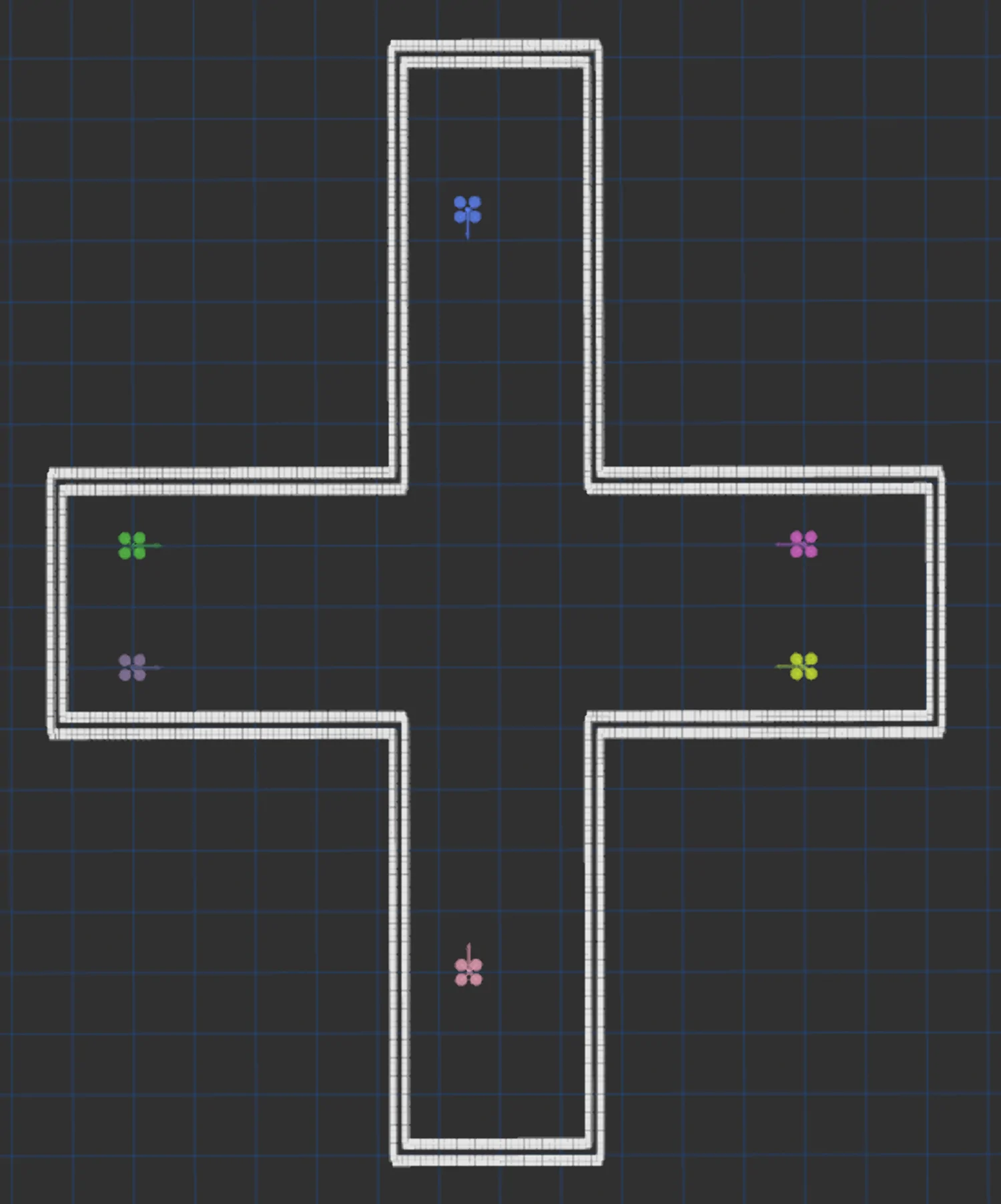
Cross-shaped environment.

**Fig 6:**
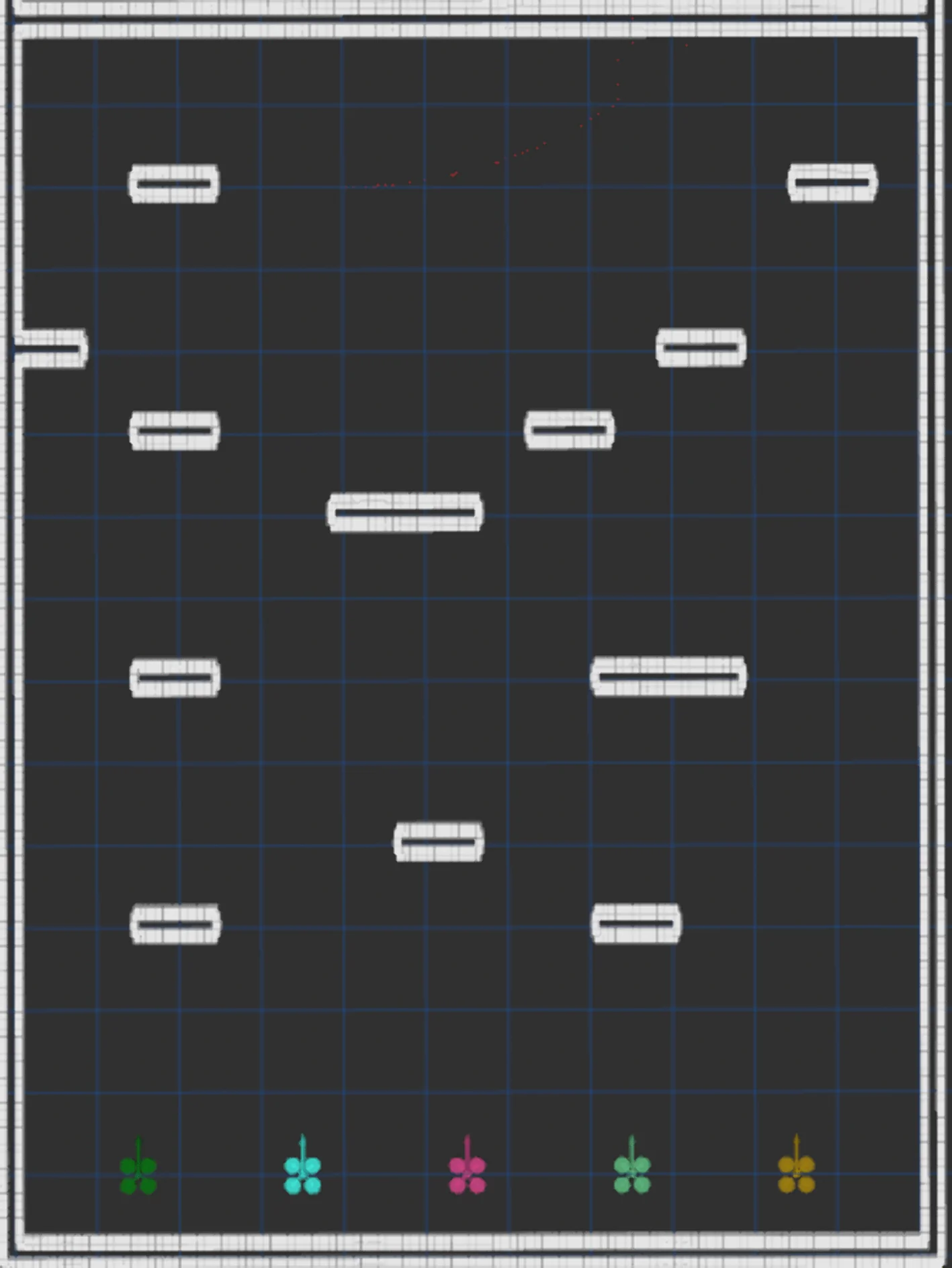
Multiple obstacles environment.

**Fig 7:**
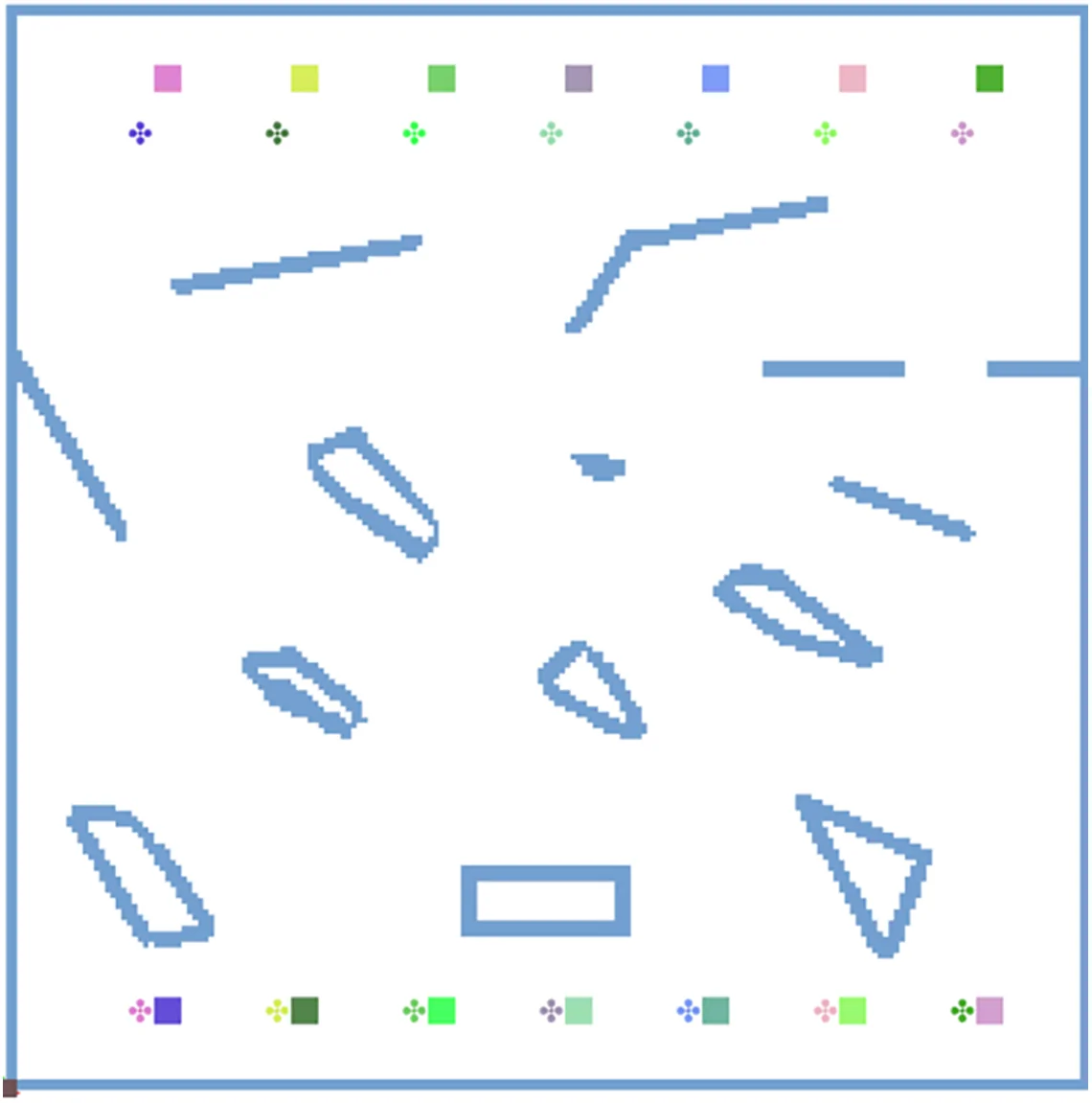
Unstructured random environment.

Three typical navigation environments of drones are constructed as simulation environment. Fig 5 is a cross-shaped environment where multiple drones need to avoid each other when moving to the center of the environment and reach the target positions on the opposite side. Fig 6 is an environment where the drones must navigate through multiple obstacles, primarily testing the obstacle avoidance capabilities of the drones. These two environments are deployed on a desktop-class system, the quadrotor models in the figure represents the initial position of the drones. Fig 7 depicts an unstructured random environment containing randomly generated static and dynamic obstacles. This scene is deployed on a simulated onboard embedded system.

### 3.2 Experimental results and comparison

#### 3.2.1 Navigation results and algorithm comparison.

**Fig 8:**
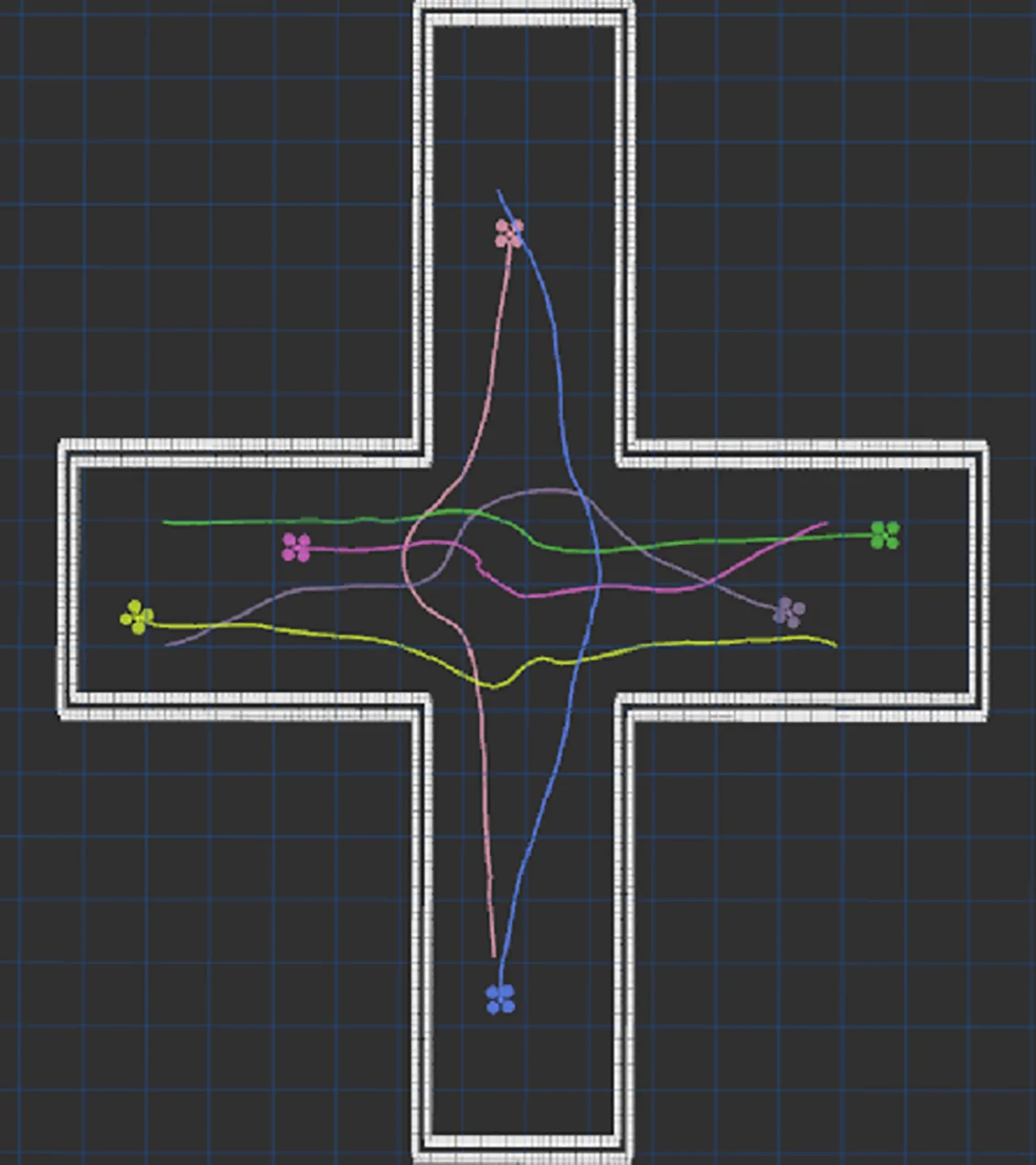
Navigation planning performance of the algorithm in cross-shaped environment.

**Fig 9:**
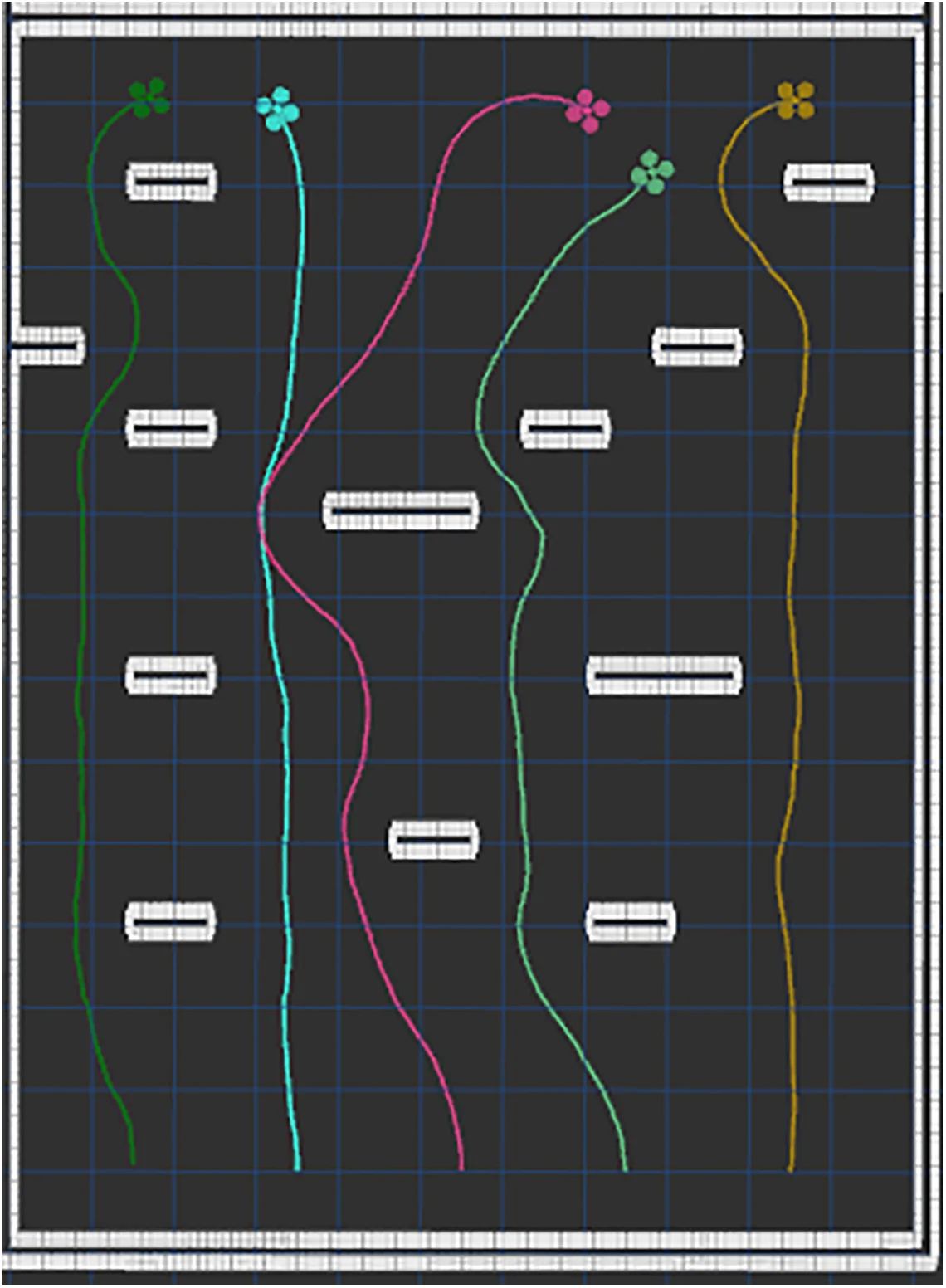
Navigation planning performance of the algorithm in multiple obstacles environment.

The navigation performance of our algorithm in the aforementioned experimental environment is shown in Figs 8 and 9. It can be observed that the algorithm effectively achieves collision avoidance between drones and navigation through multiple obstacles.

**Fig 10:**
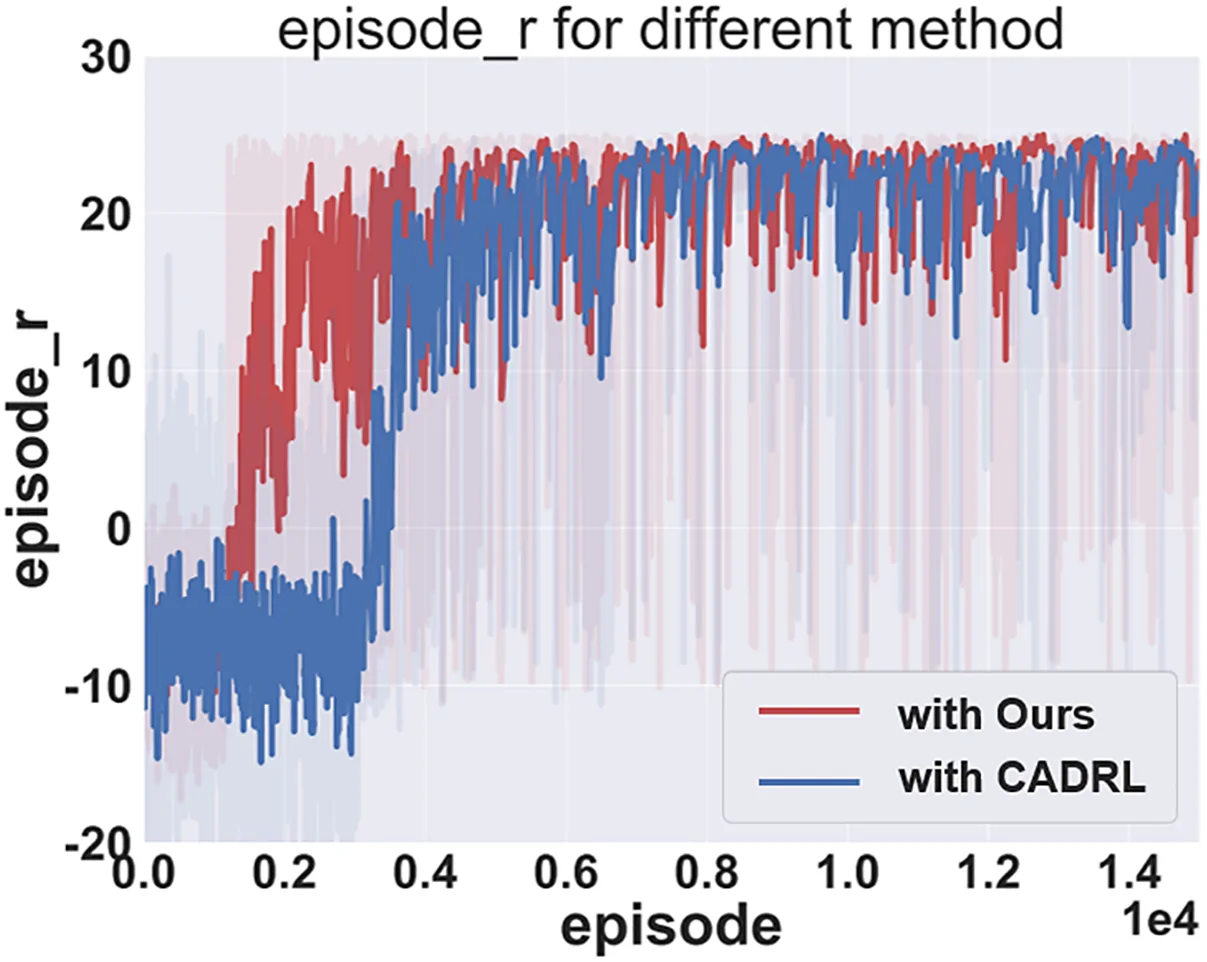
The comparative results of the reward function curve in multiple obstacles environment.

**Fig 11:**
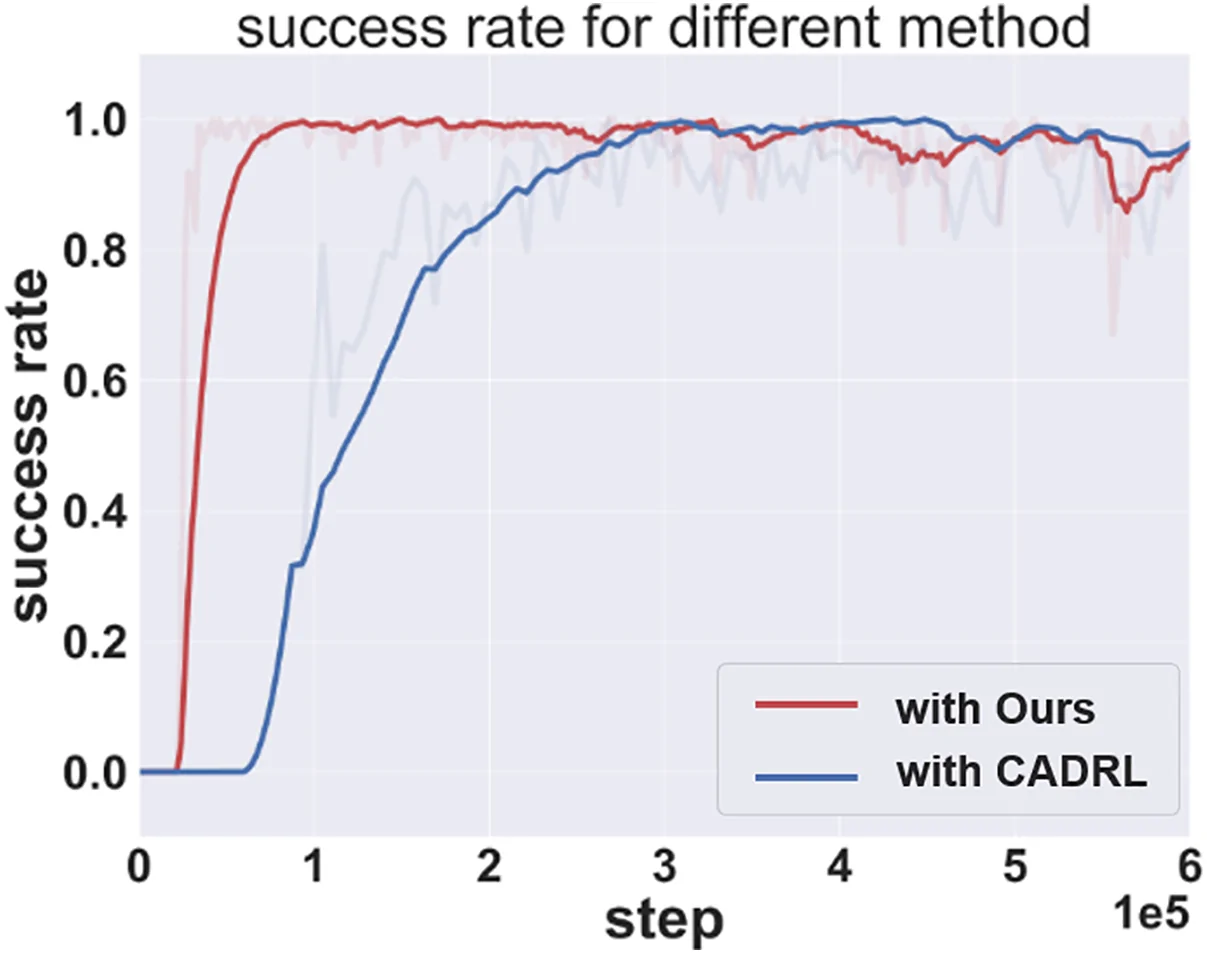
The comparative results of the success rate curve in multiple obstacles environment.

To validate the superiority of our algorithm, we conducted a comparative experiment with the Collision Avoidance with Deep Reinforcement Learning (CADRL) algorithm under the same conditions. The test scenario is the multiple obstacles environment. The experiment was configured with a fixed number of 15000 training episodes. Upon completion of each task, the cumulative reward for a single episode was collected for each drone. Additionally, the navigation success rate for all drones was tested every 3000 steps. The comparative results of the reward function curve and the success rate curve are presented in Figs 10 and 11.

The experimental results indicate that the drone swarm using our algorithm achieved a high cumulative reward after training up to 3000 episodes, whereas the CADRL algorithm required 6000 episodes to reach a comparable level. This demonstrates that our algorithm improves the convergence speed of the network by approximately a factor of two. Furthermore, our algorithm attains a higher success rate earlier compared to the CADRL algorithm.

**Fig 12:**
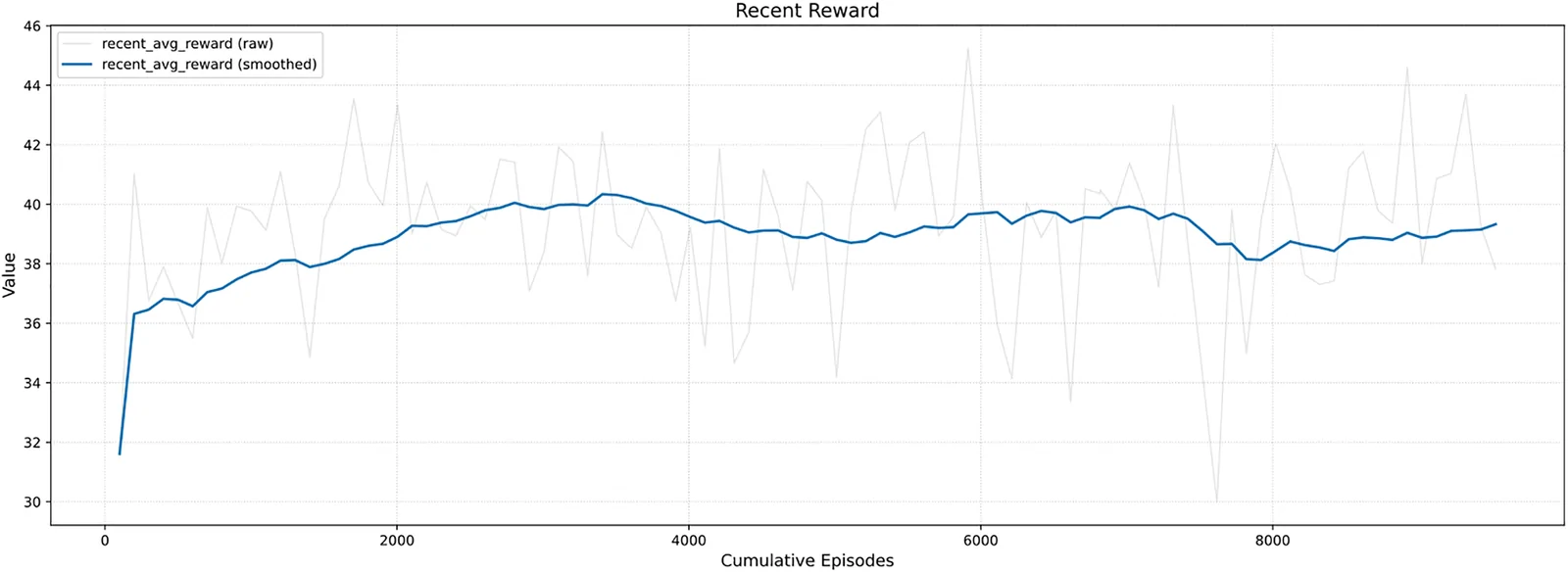
The results of the reward function curve in the unstructured random scenario.

**Fig 13:**
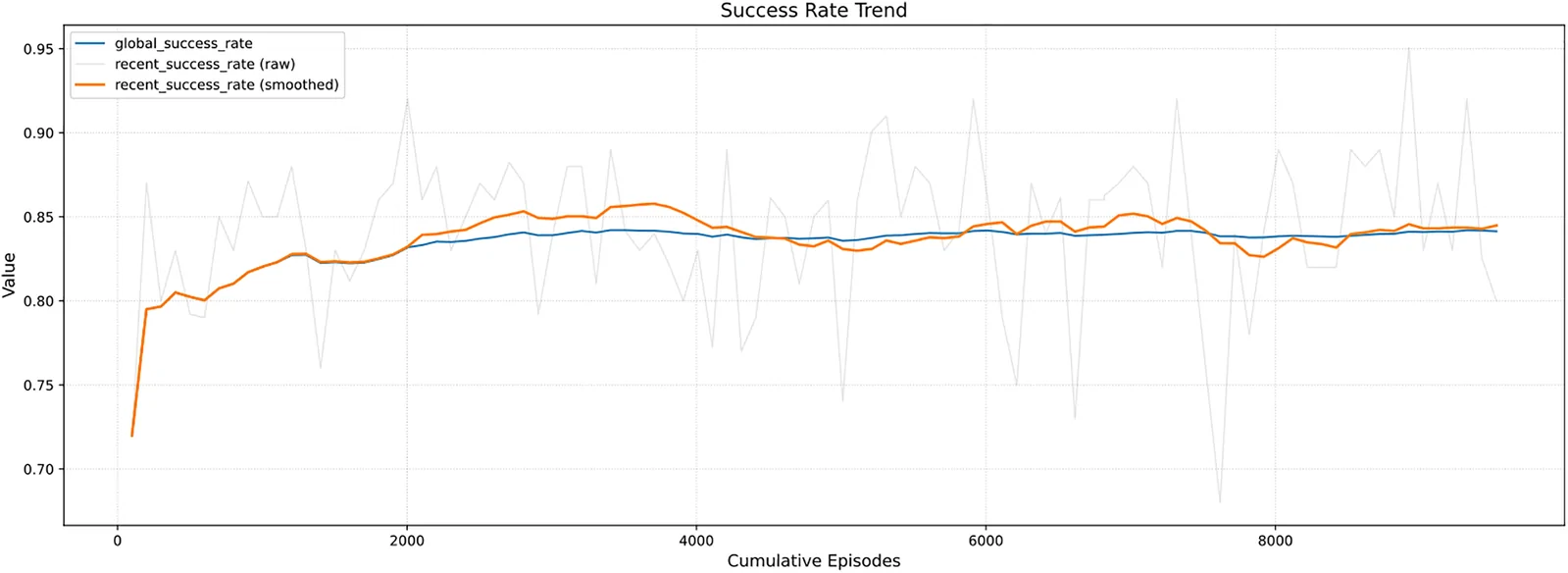
The results of the success rate curve in the unstructured random scenario.

To further explore the navigation performance of the proposed algorithm under more realistic conditions, additional validation was conducted on a simulated UAV onboard embedded system using the constructed unstructured scenario. The number of UAVs was set to 10, with starting points randomly distributed in the lower part of the map and target points randomly distributed in the upper part. The experiment was configured with a fixed number of 10000 training episodes. Upon completion of each task, the cumulative reward for a single episode was collected for each UAV, and the navigation success rate of all UAVs was tested every 3,000 steps. The reward function and navigation success rate variation results from the tests conducted in the unstructured random scenario are presented in Figs 12 and 13.

**Fig 14:**
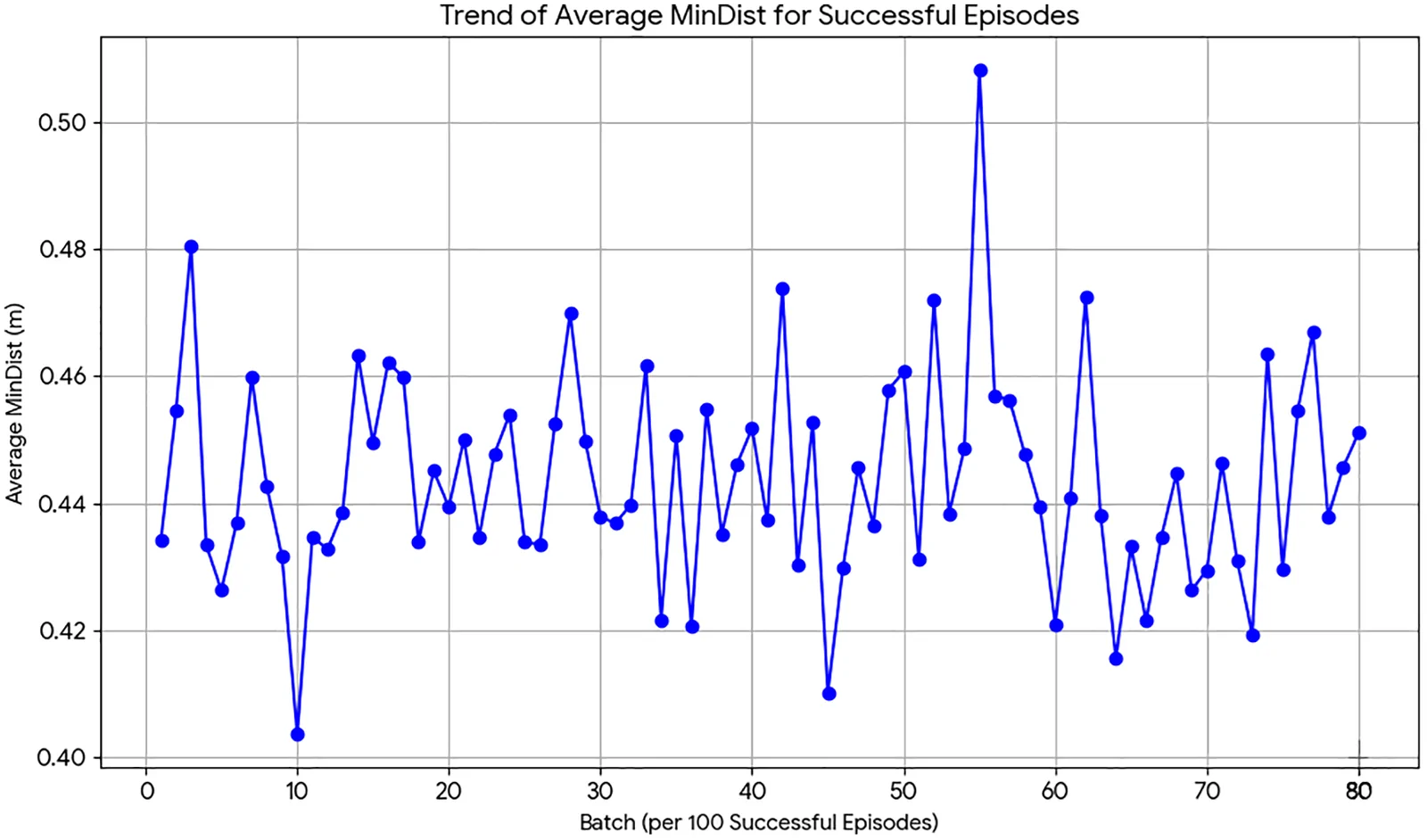
The statistical results of the minimum distance during the testing process.

As can be seen from the figure above, the algorithm began to achieve higher reward function values and success rates at approximately 150 episodes, and converged around 3,500 episodes, achieving a navigation success rate of 85%. This indicates that the algorithm can effectively adapt to unstructured scenarios and lightweight computing platforms, providing feasibility for its future application in real-world environments. We additionally collected statistics on the minimum distance between UAVs and the minimum distance between UAVs and obstacles during the testing process. Data were recorded every 100 episodes (only for successful navigation attempts), as shown in Fig 14. It can be observed that during the testing process, the minimum inter-UAV distance and the minimum distance between UAVs and obstacles remained approximately within 0.42 ~ 0.46 meters, both not falling below the safety distance of 0.4 meters, indicating that the algorithm can ensure the safety of UAVs during flight.

#### 3.2.2 Ablation studies on the improved components.

This section presents ablation studies on several key enhancements incorporated in the proposed algorithm.

- Comparative experiments of algorithms based on hierarchical planning and hybrid control strategy

The algorithms compared are: the algorithm incorporating hierarchical planning and hybrid control strategies versus the algorithm without these features. The evaluation primarily assesses success rate, timeout rate, and average reward, with the results presented in Table 1.

**Table 1:** Comparison results of algorithms based on hierarchical planning and hybrid control strategy.

Indicators	Method	Navigation environment (a)	Navigation environment (b)
success rate	with hierarchical planning and hybrid control strategy	95.4%	92.5%
	without hierarchical planning and hybrid control strategy	92.0%	90.7%
timeout rate	with hierarchical planning and hybrid control strategy	0.95%	1.13%
	without hierarchical planning and hybrid control strategy	1.25%	1.75%
average reward	with hierarchical planning and hybrid control strategy	36.15	38.56
	without hierarchical planning and hybrid control strategy	27.06	35.11

As demonstrated, the algorithm incorporating hierarchical planning and hybrid control strategies achieves a considerable improvement in navigation success rate while significantly reducing timeout instances. This indicates that the integration of a path planning-based subgoal selector and hybrid control strategy can effectively mitigate local optima issues commonly encountered in traditional RL-based navigation.

- Comparative experiments of navigation strategy with improved hybrid feature extraction network

The algorithms compared are: the algorithm incorporating the improved hybrid feature extraction network versus the algorithm without this module. The evaluation primarily focuses on success rate, timeout rate, and average reward, with the results presented in Table 2.

**Table 2:** Comparison results of navigation strategy with improved hybrid feature extraction network.

Indicators	Method	Navigation environment (a)	Navigation environment (b)
success rate	with improved hybrid feature extraction network	98.96%	93.53%
	without improved hybrid feature extraction network	87.42%	89.19%
timeout rate	with improved hybrid feature extraction network	1.02%	2.62%
	without improved hybrid feature extraction network	0.66%	1.45%
average reward	with improved hybrid feature extraction network	39.42	32.46
	without improved hybrid feature extraction network	35.25	31.09

As demonstrated, the method with hybrid feature extraction achieves a higher success rate and average reward in different navigation environment compared to the method without hybrid feature extraction. That is because the hybrid feature extraction allocates self-attention weights to the safer directions of information features, ensuring that the drones can smoothly avoid obstacles. It can also be observed that, adopting hybrid feature extraction results in a relatively higher timeout rate, which is because that in congested drone environments, this approach represents a more conservative strategy, yet it does not hinder successful obstacle avoidance.

- Comparative experiments of the dual-phase training strategy with ORCA-PSO

The algorithms compared are: the algorithm incorporating the improved dual-phase strategy with ORCA-PSO, the algorithm using the traditional ORCA-based dual-phase strategy, and the algorithm without any dual-phase strategy. The evaluation primarily assesses success rate and average reward, the average values upon the end of the first stage and upon completion of the tests are recorded separately after multiple tests. The results shown in Table 3.

**Table 3:** Comparison results of the dual-phase training strategy with ORCA-PSO.

Indicators	Method	Navigation environment (a)		Navigation environment (b)	
		Value upon the end of the first stage	Value upon the completion of tests	Value upon the end of the first stage	Value upon the completion of tests
success rate	with improved ORCA-PSO dual-phase strategy	85.62%	94.56%	82.41%	93.21%
	with traditional ORCA dual-phase strategy	69.33%	93.78%	62.60%	93.59%
	without dual-phase strategy	\	89.2%	\	88.31%
average reward	with improved ORCA-PSO dual-phase strategy	26.82	39.67	26.45	37.13
	with traditional ORCA dual-phase strategy	22.43	38.26	21.17	35.6
	without dual-phase strategy	\	35.21	\	29.65

It can be observed that by the end of the first stage, the ORCA-PSO dual-stage method proposed in this paper achieves higher reward values and success rates compared to traditional dual-stage methods. Furthermore, the final cumulative reward in the second stage surpasses the peak value reached at the end of the first stage. Simultaneously, the results indicate that, compared to methods not employing a dual-stage approach, this algorithm can achieve higher cumulative reward values and success rates more rapidly.

The above results demonstrate that the enhanced dual-phase approach can indeed accelerate strategy training. Moreover, the latter phase further enhances the generalization capability of the supervised training based on the former phase, thereby improving overall performance.

## 4 Discussion

An improved hierarchical decision-making framework for UAV swarm navigation in communication-constrained unknown environments was developed. The system architecture was designed with an upper-layer global planning module and a lower-layer autonomous navigation module. Path-node-based subgoal selection and hybrid control strategies were implemented in the upper-layer module, while the lower-layer module incorporated an enhanced neural network with hybrid feature extraction and a dual-stage training strategy combining ORCA-PSO.

Simulation experiments for autonomous UAV navigation and obstacle avoidance were performed in two distinct types of unknown complex environments. The proposed algorithm demonstrated superior performance compared to the classical CADRL algorithm across key metrics, including success rate, timeout rate, and average reward. In addition, this paper also validates the application performance of the algorithm in unstructured scenarios deployed on simulated onboard computing platforms. To evaluate the individual contributions of the three main improvements, ablation studies were conducted on: (1) the hierarchical planning and hybrid control strategy; (2) the navigation strategy with improved hybrid feature extraction network; (3) the dual-phase training strategy with ORCA-PSO. The results indicated that significant improvements in both navigation success rate and cumulative reward were achieved by the proposed method, effectively addressing obstacle avoidance and navigation requirements in unknown complex environments under communication constraints.

Three main limitations of this study should be acknowledged. First, this study investigates UAV navigation in scenarios without inter-UAV communication. Future work should incorporate communication constraint models based on practical application requirements and develop adaptive collaborative navigation methods capable of dynamically adjusting to real-time communication conditions. Second, due to computational constraints and research convenience, UAV motion was restricted to a two-dimensional plane; subsequent work could leverage high-performance computing platforms to extend sensor models and UAV mobility to three-dimensional space. Third, the validation of the proposed algorithm was conducted only on desktop-level systems and simulated onboard computing platforms. In future work, we will incorporate real data from flight controllers, LiDAR sensors, and communication interference, along with actual UAV platforms, to conduct hardware-in-the-loop simulations and real-world flight tests. This will enable a comprehensive evaluation of the algorithm’s performance under authentic conditions, thereby better addressing practical application requirements.

## Supporting information

S1 Data Data. (ZIP)
